# Rewiring NADH Metabolism Through NQO1‐Mediated Redox Cycling for Targeted Follicular Lymphoma Therapy

**DOI:** 10.1002/advs.75538

**Published:** 2026-05-07

**Authors:** Jinxing Zhang, Ziqi Wang, Ye Weng, Yulin Fu, Yuelong Jiang, Beilin Zhuang, Wenzi Zeng, Xiangchun Zhang, Bing Xu, Jie Zha, Hongjun Zhuang

**Affiliations:** ^1^ Department of Hematology School of Medicine The First Affiliated Hospital of Xiamen University and Institute of Hematology Xiamen University Xiamen China; ^2^ Key Laboratory of Xiamen for Diagnosis and Treatment of Hematological Malignancy and Xiamen Hematology Medical Quality Control Center Xiamen China; ^3^ State Key Laboratory of Tea Plant Germplasm Innovation and Resource Utilization Tea Research Institute Chinese Academy of Agricultural Sciences Hangzhou China; ^4^ Department of Endocrinology and Diabetes The First Affiliated Hospital of Xiamen University School of Medicine Xiamen University Xiamen China; ^5^ Department of Rheumatology and Clinical Immunology the First Affiliated Hospital of Xiamen University School of Medicine Xiamen University Xiamen China; ^6^ Department of Diagnostic Radiology Yong Loo Lin School of Medicine National University of Singapore Singapore Singapore

**Keywords:** copper ion, epigallocatechin gallate, follicular lymphoma, NAD(P)H: quinone oxidoreductase 1, redox cycling

## Abstract

Follicular lymphoma (FL) remains an incurable B‐cell malignancy with high relapse rates, and conventional therapies are often limited by significant toxicity, highlighting the need for novel treatments. We identified that FL exhibits markedly low expression of NAD(P)H: quinone oxidoreductase 1 (NQO1), a key enzyme required for activating quinone‐based chemotherapeutics. To overcome this, we developed a novel therapeutic strategy that simultaneously upregulates NQO1 expression and provides its quinone substrate within tumor cells. This approach leverages the dual biological function of Cu^2^^+^, which acts as both an inducer of NQO1 expression via the Nuclear factor erythroid 2‐related factor 2 (Nrf2) pathway and a catalyst for the oxidation of EGCG to its quinone form. Using a CD20‐targeted nanoplatform as a delivery tool, we achieved specific co‐delivery of EGCG and Cu^2^^+^ to follicular lymphoma cells. This strategy triggered a potent NQO1‐mediated redox cycle, resulting in severe NADH depletion and profound oxidative stress. These events activated the GADD45β‐MAPK stress‐signaling pathway, leading to mitochondrial dysfunction and apoptosis activation. In a murine FL xenograft model, this approach achieved 85% tumor growth inhibition, while maintaining a favorable safety profile. This work establishes a new therapeutic paradigm for FL, leveraging intrinsic enzyme deficiency to induce tumor‐specific, self‐amplifying cell death.

## Abstract 2

Exploiting the intrinsic NAD(P)H: quinone oxidoreductase 1 (NQO1) deficiency in follicular lymphoma, this study describes a Cluster of Differentiation 20 (CD20)‐targeted nanotherapeutic strategy to rewire reduced nicotinamide adenine dinucleotide (NADH) metabolism. By co‐delivering epigallocatechin gallate and copper ions, the platform induces NQO1 expression and catalyzes substrate formation, triggering a lethal, self‐amplifying redox cycle to induce tumor‐specific apoptosis.

## 1 Introduction

Follicular lymphoma (FL), a slow‐growing B‐cell malignancy, continues to present substantial clinical challenges due to the inability of current treatments to achieve cure, high relapse rates, and significant treatment‐associated toxicities [1, 2, 3]. Our comprehensive analysis of the Cancer Genome Atlas (TCGA) and Gene Expression Omnibus (GEO) databases revealed that FL exhibits the lowest NAD(P)H: quinone oxidoreductase 1 (NQO1) expression levels among B‐cell malignancies, establishing NQO1 deficiency as a characteristic feature of this lymphoma subtype. While the crucial role of NQO1 in mediating the anticancer activity of quinone‐based drugs has been well‐established in solid tumors [4, 5], its severe deficiency in FL presents a unique therapeutic barrier. Compounds such as doxorubicin depend on NQO1 to catalyze a two‐electron reduction that consumes NADH and initiates a redox cycle, thereby generating massive reactive oxygen species (ROS) and triggering tumor cell death [6, 7, 8]. While this mechanism enhances antitumor efficacy, it also contributes to severe off‐target toxicities, such as dose‐dependent cardiotoxicity observed with doxorubicin [9, 10, 11, 12]. Furthermore, specific NQO1‐targeting agents like β‐lapachone and napabucasin exhibit narrow therapeutic windows due to systemic toxicity [13, 14]. These constraints underscore the urgent need for novel strategies that can overcome the NQO1 deficiency in FL while minimizing adverse effects.

**SCHEME 1:**
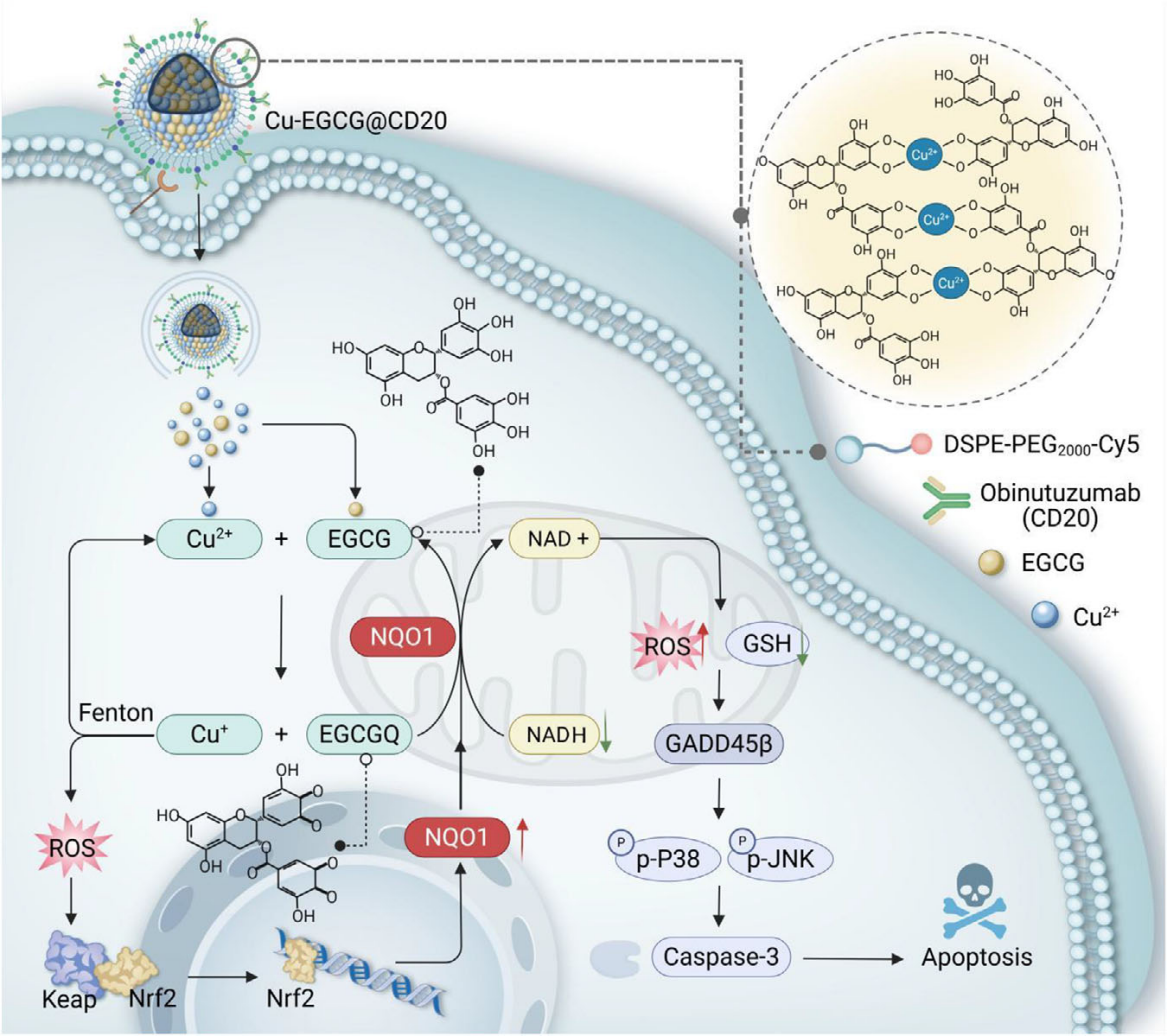
Schematic illustration of rewiring NADH metabolism through NQO1‐mediated redox cycling for targeted lymphoma therapy.

The natural polyphenol epigallocatechin gallate (EGCG) represents a promising candidate due to its favorable safety profile and multifunctional anticancer properties [15, 16, 17]. Its oxidized form, EGCG quinone (EGCGQ), can serve as an NQO1 substrate and participate in redox cycling [18, 19, 20]. However, the low basal NQO1 expression in FL cells limits the effectiveness of this approach, while the slow spontaneous oxidation of EGCG and its poor bioavailability present additional challenges [21, 22]. As shown in Scheme 1, to address the dual challenges of NQO1 deficiency and drug toxicity, we developed an innovative therapeutic strategy based on the coordination complex formed between EGCG and copper ions (Cu^2^^+^). This design leverages the dual functionality of Cu^2^^+^, which acts as an inducer of NQO1 expression via the Nrf2 pathway activation to compensate for its inherent low expression in FL cells, while also catalyzing the oxidation of EGCG to its quinone form EGCGQ.

The EGCG‐Cu^2^^+^ coordination complex was engineered into nanoparticles and surface‐functionalized with the therapeutic antibody obinutuzumab (CD20) to create Cu‐EGCG@CD20 NPs. Experimental validation demonstrated that this system effectively overcame the NQO1 deficiency in FL cells, achieving synergistic effects. The treatment triggered a cascade of metabolic events, including NADH depletion, profound oxidative stress, leading to growth arrest and DNA‐damage‐inducible protein 45 beta (GADD45β) activation and Mitogen‐activated protein kinase (MAPK) pathway phosphorylation. This ultimately resulted in mitochondrial apoptosis activation, with cleaved caspase‐3 levels increasing 4.07‐fold. In a murine FL xenograft model, the targeted nanoparticles achieved 85% tumor growth inhibition and induced apoptosis levels 9.24‐fold higher than controls, while maintaining normal serum biochemical parameters and showing no significant organ toxicity. These findings establish a dual‐pathway strategy that effectively compensates for NQO1 deficiency while minimizing off‐target effects, providing a targeted therapeutic option for FL.

## 2 Results and Discussions

### 2.1 NQO1 Deficiency in B‐Cell Non‐Hodgkin Lymphoma and Its Therapeutic Potential in Mediating the Redox Cycle

**FIGURE 1:**
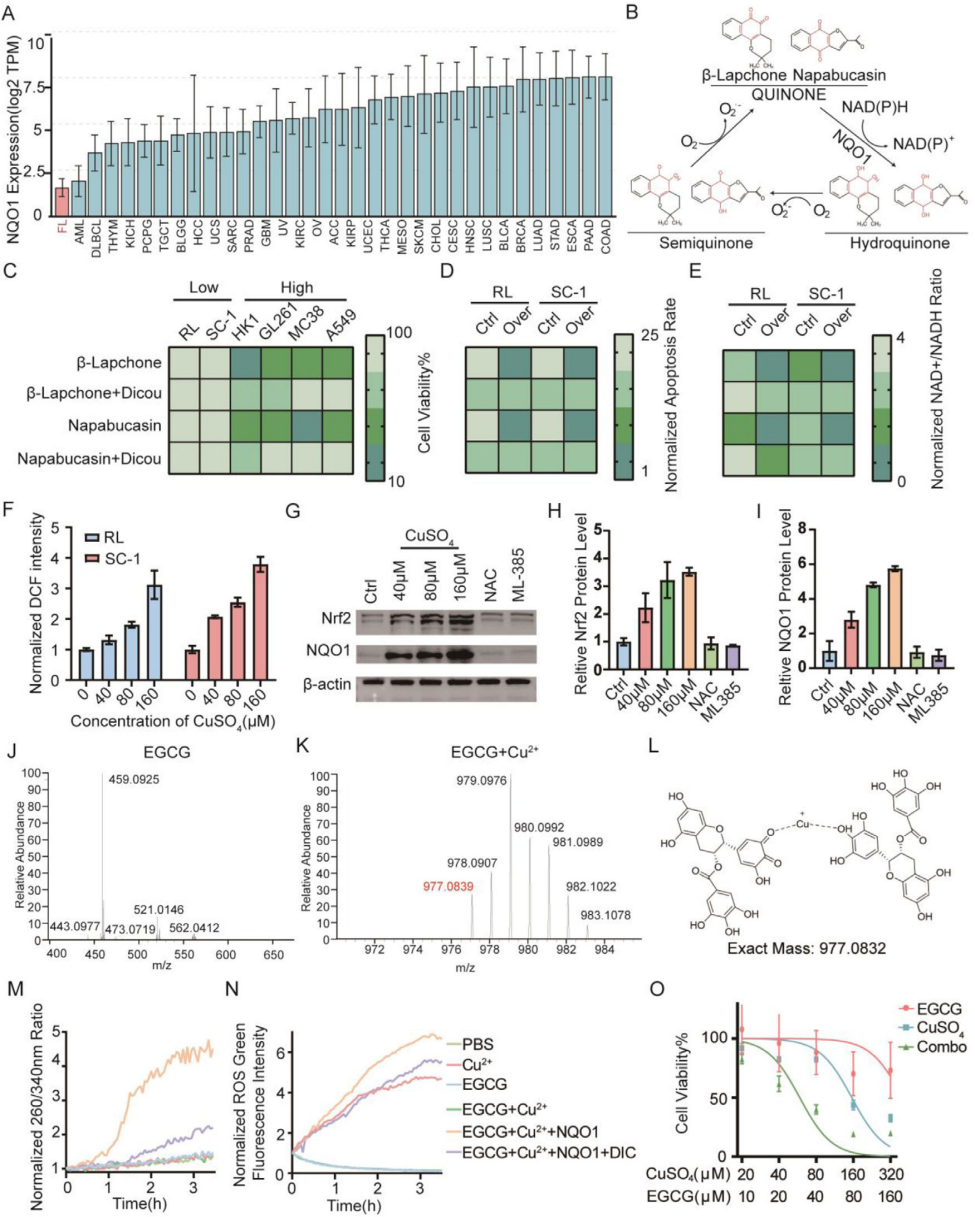
NQO1 deficiency in follicular lymphoma and the dual‐role mechanism of copper ions in therapeutic sensitization. (A) Bioinformatic analysis of NQO1 mRNA expression levels across 34 cancer types using the TCGA database and pseudo‐bulked GSE290351 dataset, identifying follicular lymphoma (FL) as a model for NQO1 deficiency. (B) Schematic representation of the NQO1‐mediated redox cycle and ROS generation induced by quinone drugs (e.g., β‐lapachone and napabucasin). (C) Cell viability (CCK‐8 assay) of FL (RL, SC‐1) and non‐FL cell lines treated with β‐lapachone or napabucasin, with or without the NQO1 inhibitor dicoumarol (DIC). (D–E) Functional validation in NQO1‐overexpressing FL cells, showing the reversal of drug sensitivity, apoptosis rates, NAD^+^/NADH ratios, and mitochondrial membrane potential by DIC.(F) Flow cytometric analysis of intracellular ROS levels in RL and SC‐1 cells treated with increasing concentrations of Cu^2^^+^ (0–160 µm). (G–I) Western blot and quantitative analysis of Nrf2 and NQO1 protein expression in FL cells following Cu^2^^+^ treatment, including reversal effects of the antioxidant NAC and Nrf2 inhibitor ML‐385. (J–L) High‐resolution mass spectrometry (HRMS) identification of the EGCG oxidation product (EGCGQ) and the proposed chemical structure of the EGCGQ‐Cu coordination complex ([M+H]^+^ = 977.0839).(M,N) Evaluation of the NQO1‐driven futile redox cycle in a cell‐free system, showing NADH consumption (260/340 nm absorbance ratio) and ROS fluorescence intensity, reversible by DIC. (O) Synergistic cytotoxicity of Cu^2^^+^ and EGCG combination treatment in FL cells measured by CCK‐8 assay after 24 h of treatment. *Quantitative data are presented as mean ± SD. Statistical significance: ^****^ *p* < 0.001, ^*^ *p* < 0.05.

A combined analysis of the TCGA database and the pseudo‐bulked GSE290351 dataset revealed that among 34 cancer types, follicular lymphoma (FL) exhibits the lowest NQO1 mRNA expression. Its expression level is approximately one‐fifth of that observed in colorectal adenocarcinoma (0.20), head and neck squamous cell carcinoma (0.23), and lung adenocarcinoma (0.21), and only about one‐third of that in glioblastoma (0.35) (Figure 1A). This establishes FL as a key model for studying NQO1‐deficient malignancies. The therapeutic significance of this finding stems from the crucial role of NQO1 in mediating the anticancer activity of quinone‐based drugs. Compounds such as β‐lapachone and napabucasin initiate cytotoxic effects through NQO1‐mediated redox cycling, which depletes NADH, generates substantial reactive oxygen species (ROS), and triggers tumor cell death (Figure 1B).

Experimental validation confirmed that FL cell lines (RL and SC‐1) exhibit low NQO1 expression at the protein level, whereas non‐FL control lines (HK1, U251, MC38, and A549) showed significantly higher expression (Figure S1A, B). Functionally, CCK‐8 assays indicated that FL cells possess lower sensitivity to quinone drugs. The inhibition rates of β‐lapachone and napabucasin were below 30% in FL cells, compared to over 40% in non‐FL cells. This difference was eliminated by the addition of the NQO1 inhibitor dicoumarol (DIC) (Figure 1C), confirming the dependence of this cytotoxicity on NQO1 expression. To further verify this relationship, we engineered NQO1‐overexpressing FL cell lines (Figure S1C). These cells exhibited markedly enhanced drug sensitivity, with inhibition rates exceeding 50%, apoptosis rates increasing by over 10‐fold, the NAD^+^/NADH ratio upregulating by more than 2.83‐fold, and mitochondrial membrane potential decreasing by over 11‐fold. Crucially, these profound effects were fully reversible by DIC (Figure 1D,E; Figure S1D,E). These results establish low NQO1 expression as the primary factor driving FL resistance to quinone drugs and highlight the simultaneous upregulation of NQO1 and provision of quinone substrates as a promising treatment strategy. Although prior studies have utilized radiotherapy or photodynamic therapy to induce NQO1, these approaches are less suitable for deep‐seated lymphomas and are often limited by the inherent toxicity of conventional quinone drugs [23, 24].

Our study introduces an innovative method utilizing the dual functionality of Cu^2+^. Flow cytometric analysis revealed that Cu^2+^ treatment induced a significant, dose‐dependent increase in intracellular ROS levels. Specifically, treatment with 40 µm Cu^2+^ elevated ROS levels by 1.31‐fold in RL cells and 2.07‐fold in SC‐1 cells, reaching maximum increases of 3.12‐fold and 3.79‐fold, respectively, at a concentration of 160 µm Cu^2+^ (Figure 1F). Concurrently, Cu^2+^ treatment induced a significant, dose‐dependent upregulation of Nrf2 and NQO1 expression. In RL cell lines, treatment with 40 µm Cu^2+^ elevated Nrf2 and NQO1 levels by 2.23‐fold and 2.80‐fold, respectively, with induction peaking at 160 µm Cu^2+^ (3.52‐fold for Nrf2 and 5.75‐fold for NQO1). This activation was significantly suppressed upon co‐treatment with the antioxidant N‐acetylcysteine (NAC) or the Nrf2 inhibitor ML‐385, which restored protein expression to near‐baseline levels. These results confirm that Cu^2+^ upregulates NQO1 through the ROS‐Nrf2 signaling pathway (Figure 1G–I).

Simultaneously, Cu^2+^ catalyzes the oxidation of EGCG to its quinone form, EGCGQ, thereby generating a suitable substrate for NQO1‐mediated cytotoxicity. We analyzed the EGCG oxidation products within a Cu^2+^ reaction system using high‐resolution mass spectrometry. At RT ≈ 0.01 min, a set of characteristic ion peaks consistent with a copper coordination complex ([M+H]^+^ = 977.0839) was detected. Based on this, we propose the formation of an EGCGQ‐Cu coordination complex with a theoretical molecular weight of 977.0832 (Figure 1J–L). In the solution system, we observed that NQO1 could catalyze the redox cycle of EGCG and Cu^2+^, during which substantial amounts of NADH were consumed. This consumption caused the 260/340 nm absorbance ratio of the system to increase to more than 5‐fold its initial value after 3 h, accompanied by a greater than 6‐fold increase in ROS fluorescence intensity. Importantly, this effect could be reversed by DIC (Figure 1M,N). These findings further demonstrate that NQO1 can drive EGCG and Cu^2+^ into a futile redox cycle, exhausting cellular NADH and generating massive ROS.

Combining Cu^2+^ and EGCG produced a potent synergistic effect, achieving over 60% growth inhibition in FL cells at concentrations where single agents showed limited efficacy (40 µM EGCG + 80 µm Cu^2+;^ Figure 1O). This synergy stems from the dual role of Cu^2^^+^: acting both as an inducer of NQO1 expression and as a catalyst for quinone formation, thereby addressing two key aspects of the therapeutic mechanism simultaneously. To further elucidate the synergistic mechanism of Cu^2^^+^ and EGCG in the presence of NQO1, and to enhance stability and bioavailability while preserving the favorable safety profiles of both components, we developed copper‐EGCG coordination nanoparticles (Cu‐EGCG NPs) [25].

### 2.2 Development and Functional Validation of a Targeted Delivery System for FL

**FIGURE 2:**
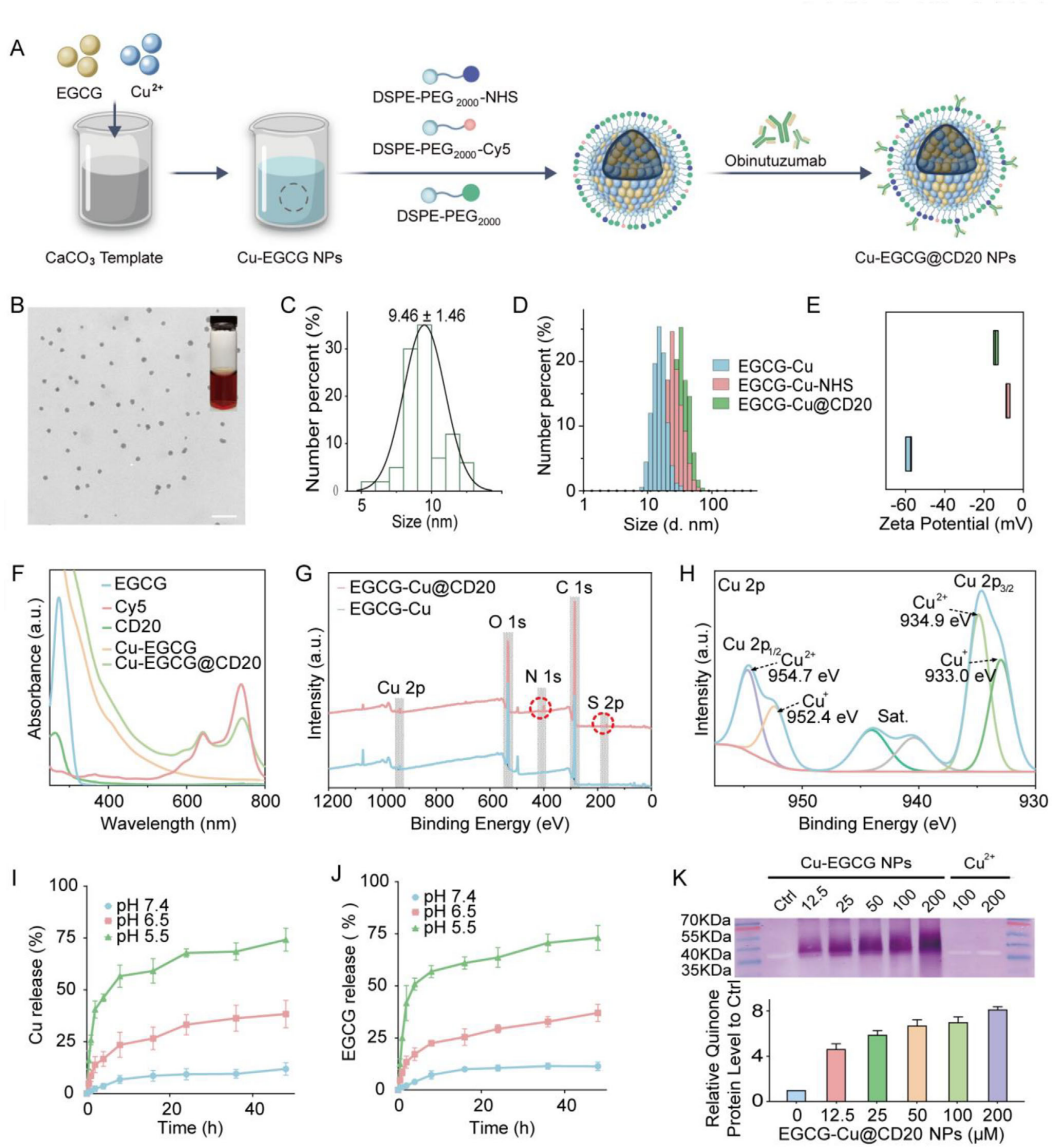
Characterization and functional analysis of Cu‐EGCG@CD20 nanoparticles. (A) Schematic diagram of the stepwise fabrication process for Cu‐EGCG@CD20 nanoparticles. (B) TEM image of synthesized nanoparticles. Scale bar: 50 nm. (C) Particle size distribution histogram from TEM analysis (n = 100 particles). (D) Hydrodynamic diameter changes during surface modification measured by dynamic light scattering. (E) Zeta potential evolution during stepwise functionalization. Data represent mean ± SD (n = 3). (F) UV‐Vis absorption spectra confirming successful integration of all components. (G) XPS survey spectrum showing elemental composition of the nanoparticles. (H) High‐resolution XPS spectra in the Cu 2p region. Cumulative release profile of Cu^2^^+^ (I) and EGCG (J) from nanoparticles at pH 5.5, 6.5 and 7.4. (K) Quinone radical generation detected by GAPDH binding assay.

We developed a targeted nanoplatform to address the therapeutic challenges identified in Section 2.1. Building upon the established coordination chemistry between copper ions and EGCG, we constructed CD20‐directed nanoparticles through sequential surface modification. The nanoparticles were functionalized with DSPE‐PEG_2000_‐NHS and the therapeutic antibody CD20, yielding Cu‐EGCG@CD20 nanoparticles (Figure 2A). This design strategy leveraged both passive targeting through enhanced permeability and retention effects and active targeting through CD20 receptor recognition. Comprehensive characterization confirmed the successful assembly of the nanoplatform. Transmission electron microscopy (TEM) revealed monodisperse spherical nanoparticles with an average diameter of 9.46 nm under physiological conditions (pH 7.4), demonstrating excellent size uniformity ideal for tumor accumulation (Figure 2B,C). Systematic analysis showed a stepwise increase in hydrodynamic diameter following each modification step (Figure 2D). Zeta potential measurements documented the expected charge evolution from strongly negative (−58.1 mV for bare Cu‐EGCG NPs) to near‐neutral (−7.5 mV after PEGylation) and finally to −9.8 mV after antibody conjugation (Figure 2E). This progressive modification pattern confirmed successful surface functionalization. Spectroscopic analyses provided molecular‐level validation of the nanoplatform's composition. Ultraviolet‐Visible (UV‐Vis) spectroscopy confirmed the integration of all components through characteristic absorption peaks (Figure 2F). X‐ray photoelectron spectroscopy (XPS) detected the expected elemental signature, including Cu, O, N, C, and S (Figure 2G; Figure S2A). High‐resolution analysis revealed the formation of mixed‐valence copper nanodots, with distinct Cu^2^^+^ (954.7/934.9 eV) and Cu^+^ (952.4/933.0 eV) signals indicating partial reduction during coordination (Figure 2H). Fourier transform infrared (FT‐IR) spectroscopy further verified successful coordination and conjugation through characteristic spectral changes (Figure S2B). Furthermore, the long‐term stability of the Cu‐EGCG@CD20 nanoparticles was thoroughly investigated. Following a 7‐day incubation in various physiological solutions—including RPMI‐1640 medium, RPMI‐1640 supplemented with fetal bovine serum (FBS), pure FBS, and phosphate‐buffered saline (PBS)—the dynamic light scattering (DLS) size and zeta potential of the nanoparticles remained essentially stable (Figure S2C,D).

Functional validation demonstrated the nanoplatform's specific targeting capability. Flow cytometry analysis showed markedly enhanced binding to CD20‐positive RL cells with 6.74‐fold increases. In contrast, CD20‐negative controls showed only a 3.85‐fold increase, confirming CD20‐dependent targeting specificity (Figure S2E,F). This antibody‐mediated targeting mechanism, combined with the modified surface properties, promoted efficient cellular internalization specifically in target cells. The nanoplatform exhibited smart release characteristics crucial for tumor‐specific therapy. Under simulated tumor microenvironment conditions (pH 5.5), both EGCG and Cu^2^^+^ showed rapid release profiles with cumulative rates exceeding 55% within 10 h. This was significantly higher than the approximately 20% release observed at physiological pH (7.4) (Figure 2I,J). The pronounced pH‐dependent release behavior ensures preferential drug release in acidic tumor tissues while minimizing systemic exposure. The development of stimuli‐responsive release systems is a key strategy for enhancing the targeted release of therapeutic agents and their biosafety. We constructed CD20‐targeting and acid‐responsive Cu‐EGCG@CD20 NPs to achieve precise intervention in follicular lymphoma [26, 27].

Solution‐phase validation confirmed the nanoplatform's functional activity. Glyceraldehyde‐3‐phosphate dehydrogenase (GAPDH) binding assays demonstrated concentration‐dependent generation of EGCG quinone products (Figure 2K), establishing the preserved ability to produce bioactive species crucial for therapeutic efficacy. This functional integrity, combined with the demonstrated targeting capability and controlled release properties, positions the Cu‐EGCG@CD20 nanoplatform as a promising therapeutic strategy for FL treatment.

### 2.3 Elucidating the Mechanism of NQO1‐Mediated NADH Depletion, Oxidative Stress, and Cell Death

**FIGURE 3:**
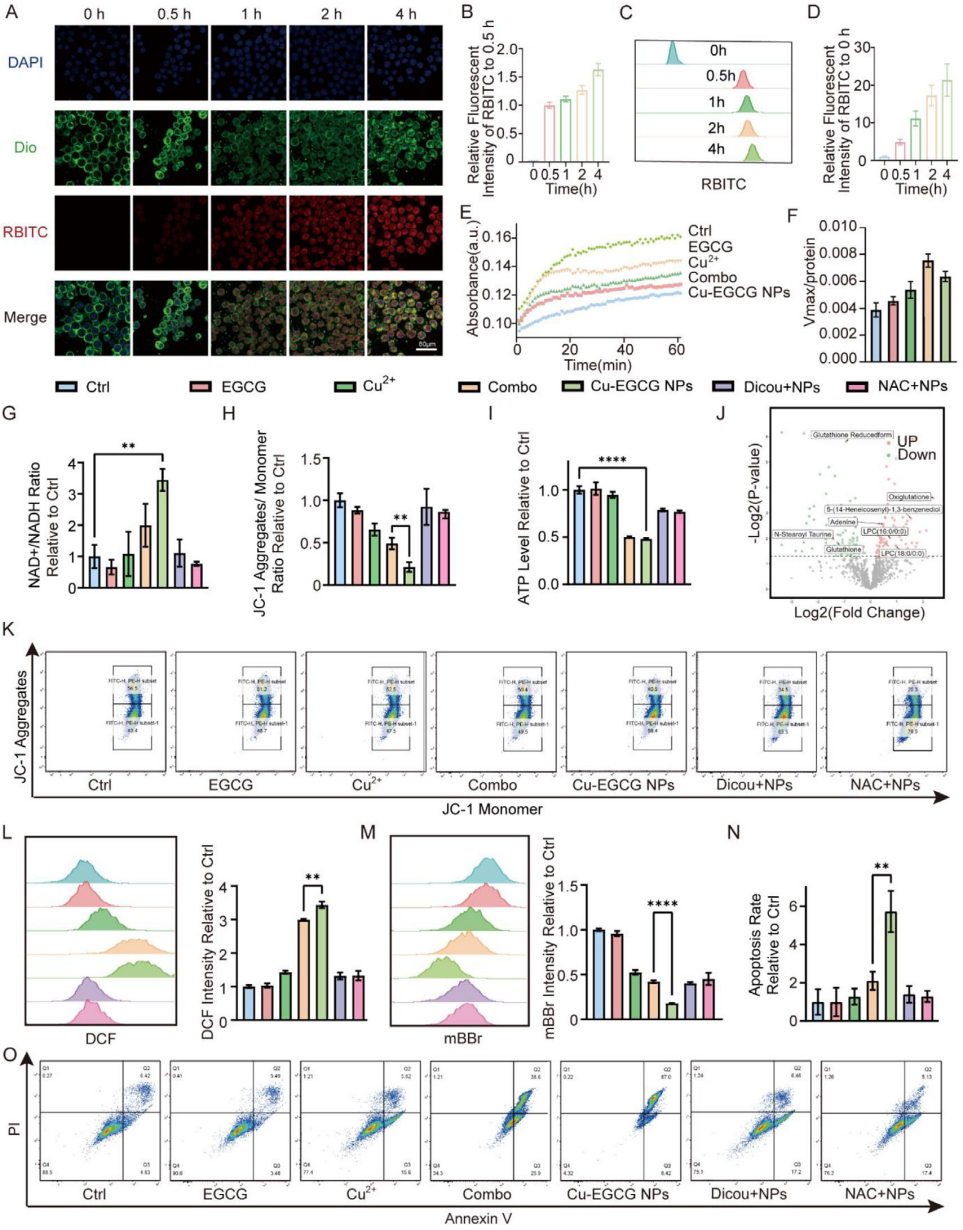
Mechanisms of Cellular Internalization, Metabolic Reprogramming, and Apoptosis Induction by Cu‐EGCG NPs. (A) Confocal images of RL cells after incubation with RBITC‐labeled (red) Cu‐EGCG NPs (cell membrane: green, DiO; nucleus: blue, DAPI). Scale bar: 80 µm. (B) Quantitative analysis of fluorescence intensity from (A). (C) Flow cytometric analysis of Cu‐EGCG NP uptake over a time course (0–4 h). (D) Quantitative analysis of the mean fluorescence intensity (MFI) from (C). (E, F) Detection of EGCGQ formation by non‐reducing SDS‐PAGE (E) and its quantitative analysis (F). (G) Effect of Cu‐EGCG NPs treatment on the intracellular NAD^+^/NADH ratio. (I) Effect of Cu‐EGCG NPs treatment on intracellular ATP levels. (H, K) Assessment of mitochondrial membrane potential (ΔΨm) by JC‐1 staining: representative flow cytometry dot plots (H) and quantitative analysis of the monomer/aggregate ratio (K). An increased ratio indicates membrane potential depolarization. (J) Volcano plot from the non‐targeted metabolomic analysis of Cu‐EGCG NPs‐treated versus control cells. (L) Intracellular ROS levels detected using the DCFH‐DA probe: representative flow cytometry histograms (left) and quantitative analysis of relative MFI (right). (M) Intracellular GSH levels detected using the mBBr probe: representative flow cytometry histograms (left) and quantitative analysis of relative MFI (right). (N, O) Apoptosis detected by Annexin V/PI double staining: representative flow cytometry dot plots (N) and quantitative analysis of the apoptosis rate (O). All quantitative data are presented as mean ± SD (n = 3). ^*^ *p* < 0.05, ^**^ *p* < 0.01, ^***^ *p* < 0.005, ^****^ *p* < 0.001 vs. the control group or other specified groups. Treatment groups include: Control (Ctrl), EGCG, Cu^2^^+^, physical mixture (EGCG + Cu^2^^+^), Cu‐EGCG NPs, Dicoumarol (Dicou) + NPs, and N‐acetylcysteine (NAC) + NPs.

We first evaluated the cellular internalization kinetics of Cu‐EGCG nanoparticles using RBITC‐labeled formulations. Confocal microscopy demonstrated time‐dependent cellular uptake, with internalization levels reaching 1.00, 1.11, 1.27, and 1.63 times baseline values at 0.5, 1, 2, and 4 h, respectively (Figure 3A,B). Flow cytometric quantification revealed a more pronounced accumulation trend, showing 4.86, 11.13, 17.25, and 21.34‐fold increases in fluorescence intensity at corresponding time points (Figure 3C,D). This discrepancy likely reflects methodological differences in detection sensitivity and cellular adhesion states. Inductively coupled plasma mass spectrometry (ICP‐MS) confirmed efficient intracellular delivery: the copper content in nanoparticle‐treated cells increased by 1.38‐fold compared to the control, whereas Cu^2^^+^‐only treatment resulted in a mere 1.13‐fold increase (Figure S3A). Functional assessment demonstrated that nanoparticle treatment enhanced EGCGQ generation by 1.63‐fold over controls (Figure 3E,F), establishing that internalized nanoparticles successfully release active components and promote quinone formation.

A key finding was the severe depletion of NADH, driven by the NQO1‐mediated redox cycle. This was reflected in the NAD^+^/NADH ratio, which surged to 3.45 times that of the control group following nanoparticle treatment (Figure 3G). This dramatic shift indicates substantial NADH consumption through the NQO1‐mediated redox cycling of EGCGQ. As NADH serves as a crucial electron donor for mitochondrial ATP production, its depletion triggered severe bioenergetic stress, reducing ATP levels to 48% of control values (Figure 3I). Concomitantly, the mitochondrial membrane potential decreased to 21.37% of the baseline (Figure 3H,K). The above effect could be effectively reversed by NAC, DIC, pyruvate supplementation (Figure S3C–F), or NQO1 siRNA (Figure S3G–J).

Metabolomic profiling revealed profound metabolic reprogramming, with clear separation between the treatment and control groups in Orthogonal Partial Least Squares‐Discriminant Analysis (OPLS‐DA) (Figure S3B). Volcano plot analysis showed a significant upregulation of oxidative stress metabolites and a marked downregulation of reductive components, including glutathione metabolites (Figure 3J). This metabolic shift reflects the compounding effects of NADH depletion and oxidative stress. The resulting oxidative burden elevated intracellular ROS levels to 3.44 times the control values, an effect that was attenuated by NAC, DIC, and treatments (Figure 3L). Concurrently, nanoparticle treatment reduced glutathione (GSH) content to 17.95% of control levels in an NQO1‐dependent manner (Figure 3M). Under this dual pressure of energy crisis and oxidative stress, the apoptosis rate increased by 5.72‐fold, an effect reversible by NAC, DIC, and NQO1 siRNA treatments (Figure 3N,O; Figure S3K,L). These findings establish a comprehensive mechanism whereby Cu‐EGCG nanoparticles trigger NQO1‐mediated NADH depletion, leading to mitochondrial dysfunction, metabolic reprogramming, and ultimately apoptotic cell death through combined energy and oxidative stress pathways.

### 2.4 Transcriptome Sequencing and Validation of the Molecular Mechanism of Apoptosis Induced by Cu‐EGCG Nanoparticles

**FIGURE 4:**
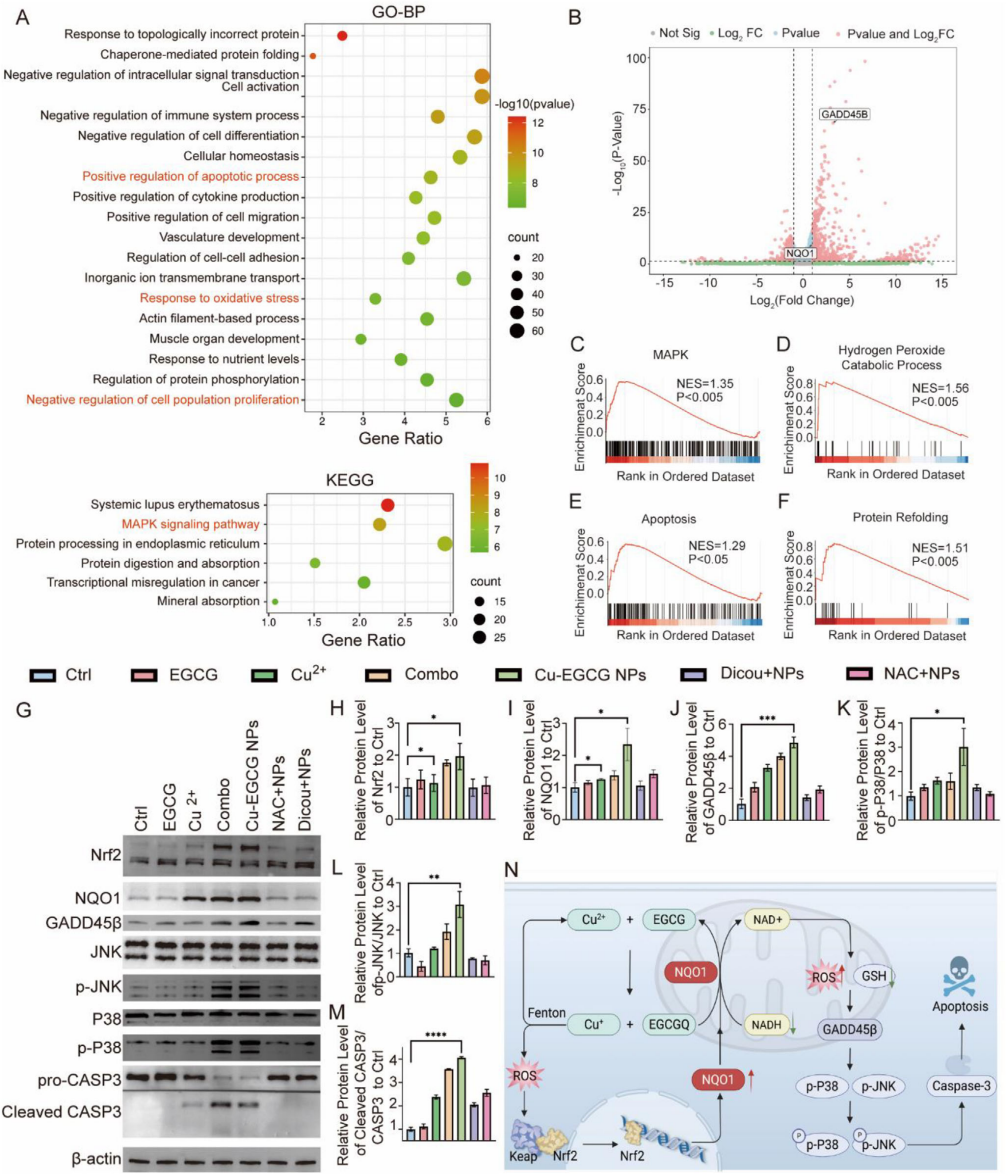
Transcriptomic analysis and mechanistic validation of Cu‐EGCG nanoparticle‐induced apoptotic pathway. (A) GO and KEGG enrichment analysis of differentially expressed genes following nanoparticle treatment. (B) Volcano plot of differentially expressed genes, highlighting key regulators (NQO1 and GADD45β). (C–F) Gene set enrichment analysis (GSEA) showing significant enrichment in (C) MAPK signaling, (D) oxidative stress response, (E) apoptosis, and (F) unfolded protein response. (G) Representative Western blot analysis of key signaling proteins across different treatment groups. (H–M) Quantitative analysis of relative protein levels or phosphorylation ratios: (H) Nrf2, (I) NQO1, (J) GADD45β, (K) p‐p38/p38 ratio, (L) p‐JNK/JNK ratio, and (M) cleaved caspase‐3/caspase‐3 ratio. (N) Schematic illustration of the proposed molecular mechanism. Panel N was created with BioRender.com. All quantitative data represent the mean ± standard deviation from three independent experiments. Statistical significance was determined by Student's t‐test (^*^ *p* < 0.05, ^**^ *p* < 0.01, ^****^ *p* < 0.001).

To comprehensively decipher the molecular mechanism by which Cu‐EGCG nanoparticles exert cytotoxic effects on follicular lymphoma cells, we conducted an integrated study combining transcriptome sequencing with functional validation. Our transcriptomic analysis revealed profound alterations in gene expression patterns. GO enrichment analysis demonstrated significant enrichment in biological processes related to apoptosis regulation, oxidative stress responses, and cell proliferation control. KEGG pathway analysis identified the MAPK signaling pathway as the most significantly enriched pathway among the differentially expressed genes (Figure 4A), providing a crucial clue into the potential mechanistic framework. Notably, we observed a substantial upregulation of NQO1 and the stress‐responsive protein GADD45β (Figure 4B), both recognized as key players in cellular stress responses. Further investigation through gene set enrichment analysis (GSEA) confirmed the significant upregulation of gene sets associated with MAPK signaling, oxidative stress responses, apoptosis execution, and the unfolded protein response (Figure 4C–F), collectively suggesting a coordinated activation of the cellular stress response.

Western blot analysis at the protein level provided compelling validation of these transcriptomic findings. Treatment with Cu‐EGCG nanoparticles significantly upregulated Nrf2 and its downstream target NQO1, with NQO1 expression showing a 2.35‐fold increase compared to the control group (Figure 4G–I). This NQO1 upregulation facilitated a redox cycling process that accelerated NADH oxidation, leading to a critical depletion of cellular NADH pools. The resulting redox imbalance triggered severe oxidative stress, evidenced by elevated ROS levels, which subsequently induced substantial activation of the stress sensor GADD45β (a 4.86‐fold increase over control levels, Figure 4J). The activated GADD45β then initiated the phosphorylation of key MAPK pathway components, as demonstrated by the p‐p38/p38 and p‐JNK/JNK ratios rising to 3.02‐fold and 3.08‐fold, respectively (Figure 4K,L). This confirms the robust activation of the MAPK pathway. These sustained phosphorylation events promoted cell apoptosis, as evidenced by the activation of the executioner caspase‐3, culminating in a 4.07‐fold increase in the cleaved caspase‐3/caspase‐3 ratio (Figure 4M).

Critical control experiments confirmed that these effects were effectively abolished by either antioxidant (NAC) or NQO1 inhibitor (dicoumarol) treatments (Figure 4N), establishing their fundamental dependence on ROS generation and NQO1‐mediated redox cycling. Furthermore, treatment with a GADD45β inhibitor (DTP3 TFA) or a combination of specific p38 (SB203580) and JNK (JNK‐IN‐8) pathway inhibitors effectively reversed the apoptotic phenotype and reduced the cleaved caspase‐3/caspase‐3 ratio by 1.80 or 2.15 fold, respectively (Figure S4A–D). This definitive blockade provides direct evidence that the activation of the GADD45β‐MAPK pathway is the terminal driver of cell apoptosis. Together, our integrated analysis establishes a comprehensive mechanistic pathway whereby Cu‐EGCG nanoparticles initiate NADH depletion through NQO1‐mediated redox cycling, leading to oxidative stress that activates the GADD45β‐MAPK axis, ultimately triggering mitochondria‐mediated, caspase‐dependent apoptosis in follicular lymphoma cells.

### 2.5 In Vivo Therapeutic Effects and Mechanisms of Cu‐EGCG@CD20 Nanoparticles in a Follicular Lymphoma Xenograft Model

**FIGURE 5:**
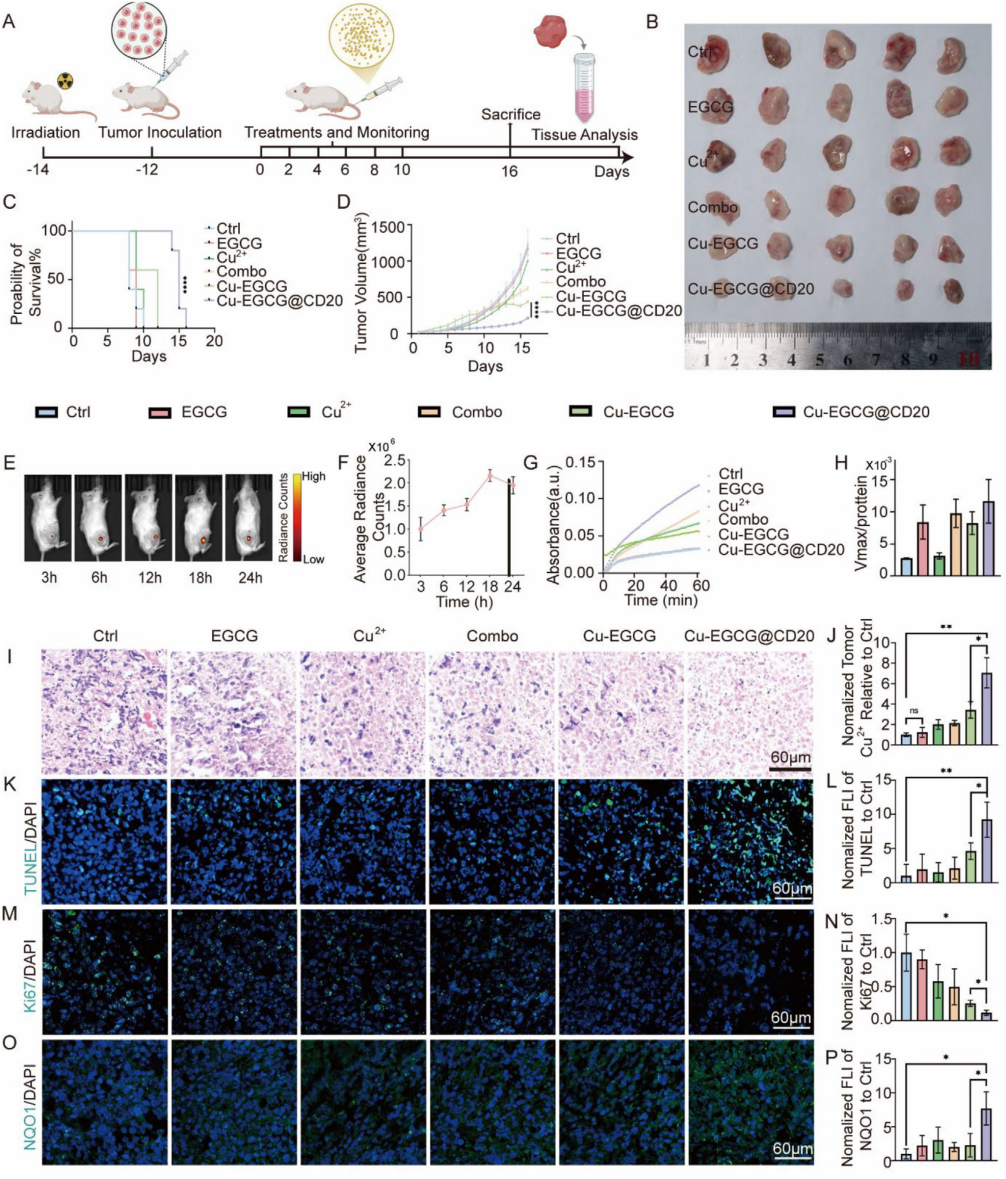
In vivo therapeutic evaluation of Cu‐EGCG@CD20 nanoparticles. (A) Experimental timeline schematic. (B) Representative tumor images at the study endpoint (day 16). (C) Kaplan‐Meier survival curves (n = 6 animals per group). (D) Tumor growth curves are presented as mean ± standard error (n = 6). (E,F) In vivo fluorescence imaging of Cy5‐labeled nanoparticles at 3–24 h post‐injection. (G,H) Quinone‐protein adduct detection and quantitative analysis. (I) H&E staining of tumor tissue sections (scale bar: 60 µm). (J) Tumor copper content quantification by Inductively Coupled Plasma Mass Spectrometry (ICP‐MS). (K,L) TUNEL staining and quantification of apoptotic cells (scale bar: 60 µm). (M,N) Ki67 staining and quantification of proliferating cells (scale bar: 60 µm). (O,P) Immunofluorescence staining of NQO1 (green) and the corresponding quantitative analysis of NQO1 fluorescence intensity (scale bar: 60 µm).All treatments were administered intravenously at 10 mg/kg Cu^2^^+^ or 36 mg/kg EGCG equivalent dosage.

Building upon our in vitro demonstration of successful CD20‐targeted nanoparticle construction, we proceeded to systematically evaluate the in vivo antitumor efficacy of Cu‐EGCG@CD20 nanoparticles. We established a subcutaneous follicular lymphoma model in mice by inoculating lymphoma cells following a single 1.2 Gy irradiation protocol. Treatment commenced 12 days after tumor nodule formation (designated as Day 0), with tail vein administration every two days for a total of six doses at a copper equivalent of 10 mg/kg (Figure 5A). Tumor tissues were harvested after 16 days of treatment for comprehensive analysis (Figure 5B). The Cu‐EGCG@CD20 nanoparticle treatment demonstrated significant therapeutic benefits. Kaplan‐Meier survival analysis revealed that while the control group reached 100% mortality by day 10, the nanoparticle‐treated group maintained 100% survival through day 13, indicating a substantial extension of survival (Figure 5C). Concurrently, nanoparticle treatment effectively suppressed tumor growth throughout the treatment period (Figure 5D). In vivo fluorescence imaging tracked the biodistribution of Cy5‐labeled nanoparticles, revealing specific accumulation in tumor regions. The targeted nanoparticle group exhibited peak signal intensity at 18 h post‐injection (Figure 5E,F; Figure S5A). This targeted accumulation translated into enhanced therapeutic effects: compared to other groups, the Cu‐EGCG@CD20 nanoparticle group showed a 7.06‐fold increase in copper content (Figure 5J) and significantly elevated quinone‐protein adduct levels (Figure 5G,H). Histopathological examination confirmed the superior efficacy of the targeted nanoparticles. H&E staining revealed the most substantial tumor architecture disruption in the Cu‐EGCG@CD20 nanoparticle group (Figure 5I). TUNEL staining showed apoptosis levels 9.24‐fold higher than controls (Figure 5K,L), while Ki67 immunohistochemistry demonstrated marked proliferation inhibition, with only 11.49% positivity in the treatment group versus controls (Figure 5M,N). NQO1 immunohistochemistry further confirmed target engagement, showing 7.71‐fold higher expression in treated tumors (Figure 5O,P).

Critically, this potent antitumor efficacy was achieved without significant safety concerns. H&E staining of major organs revealed no apparent pathological damage (Figure S5B), and serum biochemical parameters (BUN, Cr, CK, ALT, AST) remained within normal ranges (Figure S5C). Furthermore, quantitative analysis revealed that the copper ion concentration enriched in tumors was 9.34 times that of the control group, while the copper concentration in the liver was only 1.53 times that of the control. No significant differences in copper concentrations were observed in the heart, lungs, spleen, or kidneys (Figure S5D), confirming the favorable biosafety profile and targeted accumulation of this nanotherapeutic approach. These results demonstrate that Cu‐EGCG@CD20 nanoparticles achieve efficient targeted delivery to follicular lymphoma tissues, inducing NQO1 expression, promoting quinone radical generation, and activating apoptotic pathways to exert significant antitumor effects while maintaining an excellent safety profile.

## 3 Conclusion

This study presents a novel therapeutic strategy that overcomes the challenge of NQO1 deficiency in follicular lymphoma by leveraging the dual functionality of copper ions. Our approach simultaneously upregulates NQO1 expression through Cu^2^^+^‐induced Nrf2 pathway activation and generates its quinone substrate via catalytic oxidation of EGCG, creating a self‐amplifying cytotoxic cycle within tumor cells. The CD20‐targeted delivery system demonstrated high targeting specificity, with 2.16‐fold enhanced tumor accumulation. Mechanistic investigations revealed that this strategy triggers a cascade of metabolic disruptions, including NADH depletion (NAD^+^/NADH ratio increased to 0.935), oxidative stress (ROS levels elevated 3.44‐fold), and activation of the GADD45β‐MAPK pathway (GADD45β increased 4.86‐fold). These events culminated in mitochondrial apoptosis with a 4.07‐fold increase in caspase‐3 cleavage. In vivo evaluation demonstrated potent antitumor efficacy, achieving 85% tumor growth inhibition, while maintaining excellent safety. The treatment induced apoptosis rates 9.24‐fold higher than controls, confirming specific anti‐lymphoma activity. Although this study demonstrates potent efficacy in murine cell‐line xenograft models, the lack of validation in patient‐derived xenograft (PDX) models represents a limitation. Future investigations utilizing stable NQO1‐deficient FL PDX cohorts will be essential to further evaluate the clinical translational potential of this strategy. Our work establishes a new paradigm for treating enzyme‐deficient cancers by orchestrating a coordinated therapeutic circuit that addresses both enzyme expression and substrate availability. This innovative approach offers a promising direction for targeted cancer therapy that exploits metabolic vulnerabilities while minimizing systemic toxicity.

## 4 Experimental Section/Methods

### 4.1 Chemicals and Materials

Epigallocatechin gallate (EGCG, purity ≥95%) and copper sulfate pentahydrate (CuSO_4_·5H_2_O, purity ≥99%) were purchased from Sigma–Aldrich (Shanghai, China). DSPE‐PEG_2000_‐NHS and Cy5 fluorescent dye were obtained from Ruixi Biological Co., Ltd. (Xi'an, China). The anti‐CD20 monoclonal antibody (Obinutuzumab) was sourced from Selleck Chemicals (Houston, USA). Cell culture media, including RPMI‐1640 and IMDM, as well as fetal bovine serum (FBS), were supplied by Gibco (Grand Island, USA). All chemical reagents were of analytical grade and used without further purification. The Liquid Sample ROS Detection Kit (BBoxiProbe O11, bright orange fluorescence, Cat# BB‐46151‐100T) was obtained from BestBio (China). NADH (Cat# ST358), recombinant NQO1 enzyme (Cat# P2395L), and 96‐well transparent flat‐bottom UV plates (Cat# FUV961) were purchased from Beyotime Biotechnology (China). Dicoumarol (Cat# HY‐N0645) was acquired from MedChemExpress (MCE).

### 4.2 Material Characterization

The morphology and size distribution of nanoparticles were characterized using transmission electron microscopy (TEM, Hitachi HT7800, Japan) operating at 100 kV. Hydrodynamic diameter and zeta potential were measured by dynamic light scattering (DLS) using a Malvern Zetasizer Nano ZS instrument (Malvern Panalytical, UK). UV‐Vis absorption spectra were recorded on a Shimadzu UV‐2600 spectrophotometer (Kyoto, Japan). Fourier transform infrared (FTIR) spectra were obtained using a Thermo Scientific Nicolet iS50 spectrometer (Massachusetts, USA) with the attenuated total reflection (ATR) mode. The chemical composition and elemental states were analyzed by X‐ray photoelectron spectroscopy (XPS) on a Thermo Fisher Escalab Xi+ system (Massachusetts, USA) with monochromatic Al Kα radiation.

### 4.3 Preparation of Cu‐EGCG Nanoparticles

Cu‐EGCG nanoparticles were synthesized by mixing 10 mm EGCG and 5 mm CuSO_4_·5H_2_O in deionized water under constant stirring (500 rpm) at room temperature for 30 min. The resulting nanoparticles were collected by centrifugation at 12 000 × g for 10 min and washed three times with deionized water to remove unreacted precursors.

### 4.4 Surface Modification with DSPE‐PEG2000‐NHS

The obtained Cu‐EGCG nanoparticles (4 mm) were incubated with 2 mg of DSPE‐PEG_2000_‐NHS in 50 mM HEPES buffer (pH 7.4) for 30 min at room temperature with gentle agitation. The PEGylated nanoparticles (Cu‐EGCG‐PEG) were purified by centrifugation at 12 000 × g for 10 min and resuspended in HEPES buffer for subsequent conjugation.

### 4.5 Antibody Conjugation

The CD20 antibody (Obinutuzumab, 50 µg) was added to the Cu‐EGCG‐PEG solution and allowed to react for 1.5 h at room temperature. The resulting Cu‐EGCG@CD20 nanoparticles were collected by centrifugation and stored in phosphate‐buffered saline (PBS, pH 7.4) at 4°C until use.

### 4.6 Cell Culture and Treatment

Human follicular lymphoma cell lines (RL and SC‐1) and mouse myeloma cell line (5TGM1) were obtained from the American Type Culture Collection (ATCC, USA). RL and Karpas422 cells were maintained in RPMI‐1640 medium, while 5TGM1 cells were cultured in IMDM medium. All media were supplemented with 10% (v/v) heat‐inactivated FBS and 1% penicillin/streptomycin (100 U/mL penicillin and 100 µg/mL streptomycin). Cells were incubated at 37°C in a humidified atmosphere containing 5% CO_2_.

### 4.7 RNA Interference and Cell Transfection

To knock down NQO1 expression, human NQO1‐specific small interfering RNA (siRNA) was utilized. The specific sequence targeting human NQO1 (designated as NQO1 siRNA) is 5'‐GGUUUGAGCGAGUGUUCAU‐3'. The siRNA was synthesized by Sangon Biotech (Shanghai, China). For transfection, FL cells were seeded in culture plates and transfected with the NQO1 siRNA using Lipofectamine 2000 (Vazyme, Cat # TL201‐01/02, China) according to the manufacturer's protocol. Subsequent nanoparticle treatments and functional assays were performed 48 h post‐transfection.

### 4.8 Cellular Uptake Analysis

Cells were seeded in 6‐well plates at a density of 2×10^5^ cells per well and incubated with RBITC‐labeled nanoparticles (equivalent to 20 µM EGCG) for 0, 1, 2, and 4 h. Cellular uptake was quantified by flow cytometry (CytoFLEX LX, Beckman Coulter, USA) and visualized using confocal laser scanning microscopy (Leica TCS SP8, Germany) after staining with DiO (cell membrane) and DAPI (nuclei).

### 4.9 Intracellular Metal Ion Detection

Following nanoparticle treatment, cells were collected and digested with concentrated nitric acid. Intracellular copper content was determined by inductively coupled plasma mass spectrometry (ICP‐MS, Thermo Fisher iCAP RQ, USA) following appropriate dilution with ultrapure water.

### 4.10 ROS and GSH Detection

Intracellular ROS levels were measured using the fluorescent probe DCFH‐DA (2',7'‐dichlorodihydrofluorescein diacetate). GSH content was determined using monobromobinane (mBBr) staining. After incubation with the respective probes, cells were analyzed by flow cytometry.

### 4.11 Apoptosis and Mitochondrial Membrane Potential Assay

Apoptosis was evaluated using an Annexin V‐FITC/PI apoptosis detection kit (Beyotime, China) according to the manufacturer's instructions. Mitochondrial membrane potential was assessed using JC‐1 staining and analyzed by flow cytometry.

### 4.12 NAD^+^/NADH Ratio Assay

The intracellular NAD^+^/NADH ratio was determined using an NAD^+^/NADH Assay Kit (WST‐8 method, Beyotime Biotechnology, China, Cat# S0175) following the manufacturer's protocol. Briefly, cells were lysed and the lysates were processed to separately quantify NAD^+^ and NADH levels based on the WST‐8 chromogenic reaction. The absorbance was measured using a microplate reader, and the NAD^+^/NADH ratio was calculated accordingly.

### 4.13 Western Blot Analysis

Total proteins were extracted using RIPA lysis buffer containing protease and phosphatase inhibitors. Protein concentrations were determined by BCA assay. Equal amounts of protein (30 µg per lane) were separated by 10% SDS‐PAGE and transferred to PVDF membranes. After blocking with 5% non‐fat milk, membranes were incubated with primary antibodies against Nrf2, NQO1, GADD45β, phospho‐JNK, JNK, phospho‐p38, p38, and Cleaved Caspase‐3 (Proteintech, 1:1000 dilution) overnight at 4°C. HRP‐conjugated secondary antibodies and an ECL detection system (Bio‐Rad, USA) were used for signal visualization.

### 4.14 Tumor Model Establishment

Female NCG mice (4–6 weeks old) were subcutaneously inoculated with 5×10^6^ RL cells in the right flank. All animal procedures were approved by the Animal Care Committee of Xiamen University (Approval number: XMULAC20220270) and conducted in accordance with institutional guidelines.

### 4.15 Drug Administration and Monitoring

Mice were randomly divided into six groups (n = 6 per group): Control, EGCG, Cu^2^^+^, Combination (EGCG+Cu^2^^+^), Cu‐EGCG NPs, and Cu‐EGCG@CD20 NPs. Treatments were administered via tail vein injection (10 mg/kg Cu^2^^+^ equivalent) every two days for a total of six doses. Tumor dimensions and body weight were measured every two days. Tumor volume was calculated using the formula: Volume = (Length × Width^2^)/2.

### 4.16 Fluorescence Imaging

Cy5‐labeled nanoparticles were injected via the tail vein, and fluorescence signals were monitored at 3, 6, 12, and 24 h post‐injection using an IVIS Lumina XR imaging system (PerkinElmer, USA). Regions of interest (ROIs) were drawn around the tumor sites for signal quantification.

### 4.17 Histopathological Analysis

Tumors and major organs (heart, liver, spleen, lung, and kidney) were harvested, fixed in 4% paraformaldehyde, and embedded in paraffin. Sections (4 µm thickness) were stained with hematoxylin and eosin (H&E) for general morphology. Apoptosis was detected by TUNEL assay (Beyotime, C1086), and immunohistochemistry was performed for Ki67 (Beyotime, C2317S) and NQO1 expression analysis.

### 4.18 Cell‐Free Redox Cycle and ROS Generation Assays

The NQO1‐mediated redox cycling, concurrent NADH consumption, and ROS generation were evaluated in cell‐free systems. For the ROS generation assay, experiments were conducted in a BeyoGold all‐black 96‐well plate. Each reaction was carried out in a total volume of 300 µL. The standard reaction system consisted of 200 µL of the ROS probe, 10 µL of NADH (5 mM stock), and 5 µL of recombinant NQO1. Based on the designated experimental groups (Control, EGCG alone, Cu^2^^+^ alone, Cu^2^^+^+EGCG, and Dicoumarol rescue), specific volumes of EGCG (4 µL of 2.5 mm stock), Cu^2^^+^ (4 µL of 5 mM stock), and Dicoumarol (4 µL) were supplemented. An NQO1‐free group was also included as a negative control. The final volume for all reactions was uniformly adjusted to 300 µL using PBS. For the NADH consumption assay, the experimental design and reagent volumes remained identical, except that the reactions were performed in a 96‐well transparent flat‐bottom UV plate, and the 200 µL of ROS probe in each well was replaced with 200 µL of PBS. To prevent liquid evaporation during continuous monitoring, all empty wells and the inter‐well gaps of the microplates were filled with PBS. Measurements were performed using a Tecan Spark multimode microplate reader. NADH consumption was tracked by monitoring the absorbance ratio at 260/340 nm, while ROS generation was quantified by measuring the bright orange fluorescence intensity of the BBoxiProbe O11.

### 4.19 Statistical Analysis

All data are presented as mean ± standard deviation (SD). Statistical significance was determined using Student's t‐test for comparisons between two groups, or one‐way ANOVA followed by Tukey's post‐hoc test for multiple group comparisons using GraphPad Prism 8.0 software. A p‐value < 0.05 was considered statistically significant.

## Conflicts of Interest

The authors declare no conflicts of interest.

## Supporting information




**Supporting File**: advs75538‐sup‐0001‐SuppMat.docx.

## Data Availability

The data that support the findings of this study are available from the corresponding author upon reasonable request.
