# Supplementary material for: Rewiring NADH Metabolism Through NQO1‐Mediated Redox Cycling for Targeted Follicular Lymphoma Therapy
Source: Adv Sci (Weinh). 2026 May 7;13(42):e75538. doi: 10.1002/advs.75538 (PMC13335828; doi:10.1002/advs.75538)
Supplement: Supplementary file 1 — Supporting File: advs75538‐sup‐0001‐SuppMat.docx. [file ADVS-13-e75538-s001.docx]

Supporting Information

1. Additional Figures and Tables

**
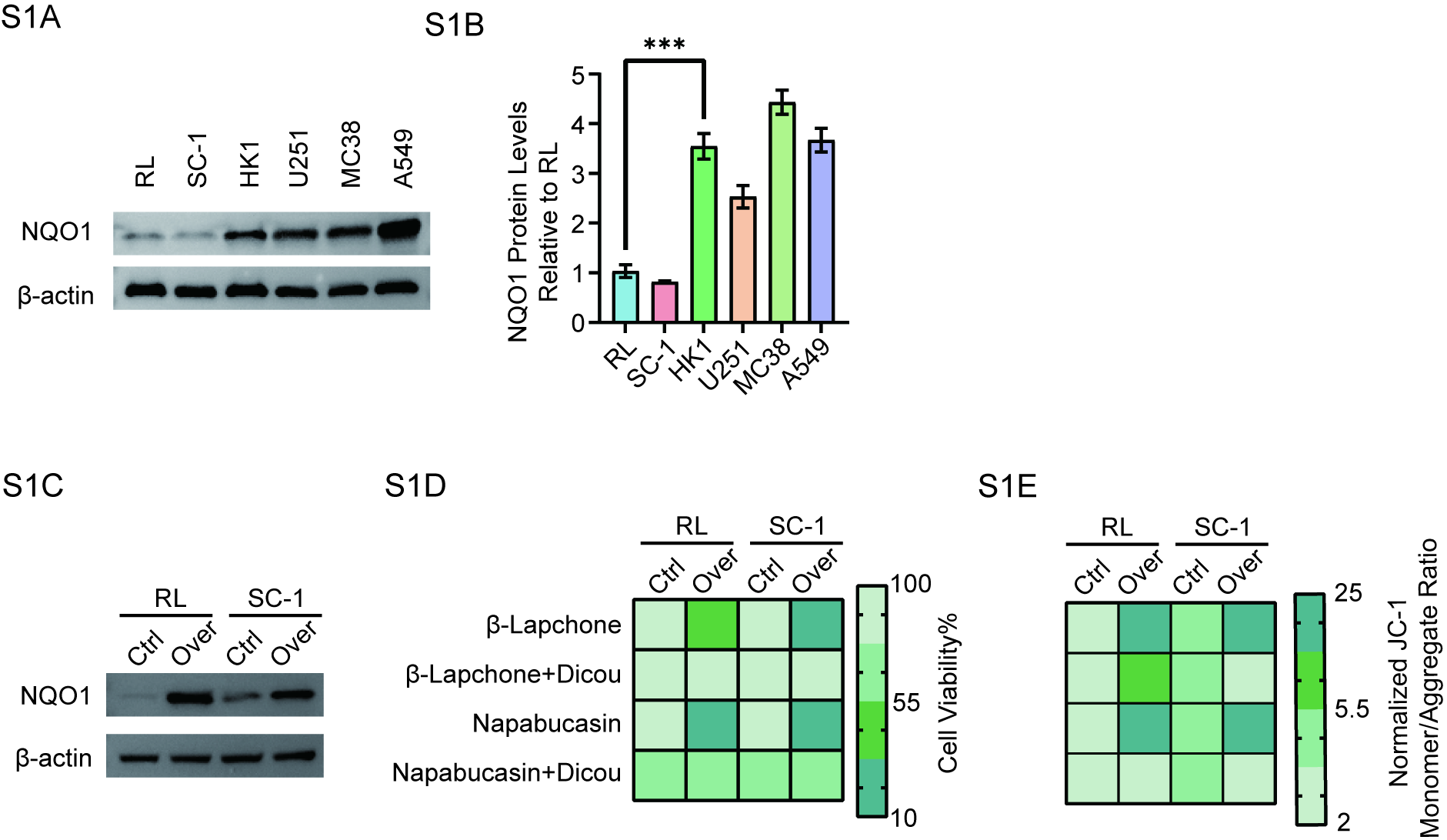
**

**Supplementary Figure 1. NQO1 expression profiles and in vitro functional validation of NQO1 overexpression in follicular lymphoma cells.**(A, B) Western blot analysis (A) and corresponding quantitative statistical analysis (B) of NQO1 protein expression levels in follicular lymphoma (FL) cell lines (RL, SC-1) compared to non-FL control cell lines (HK1, U251, MC38, A549). (C) Western blot bands confirming the successful engineering and expression of NQO1 in NQO1-overexpressing (NQO1-OE) RL and SC-1 cell lines. (D) Cell viability measured by CCK-8 assay, demonstrating the enhanced sensitivity of NQO1-overexpressing FL cells to quinone drugs compared to wild-type controls. (E) Assessment of mitochondrial membrane potential using JC-1 staining in control and NQO1-overexpressing FL cell lines following quinone drug treatment.


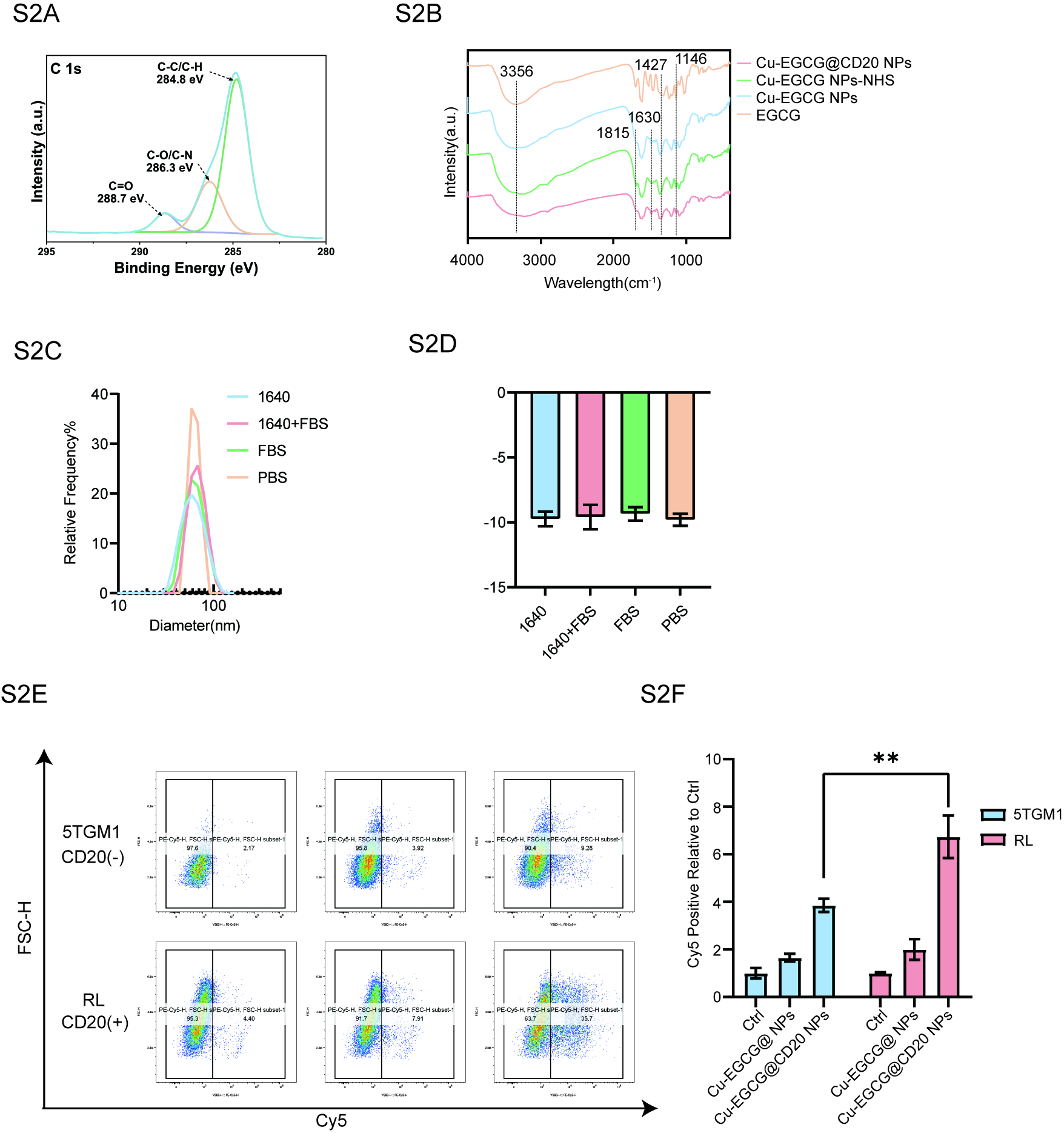


**Supplementary Figure 2. Supplemental characterization, stability, and CD20-targeted binding capability of Cu-EGCG@CD20 nanoparticles.**(A) X-ray photoelectron spectroscopy (XPS) spectra detailing the elemental signature (Cu, O, N, C, and S) of the Cu-EGCG@CD20 nanoparticles. (B) Fourier transform infrared (FT-IR) spectra verifying the successful coordination and stepwise surface conjugation of the nanoparticles. (C, D) Long-term stability assessment showing the dynamic light scattering (DLS) size distribution (C) and zeta potential (D) of Cu-EGCG@CD20 nanoparticles following a 7-day incubation in RPMI-1640 medium, RPMI-1640 + FBS, pure FBS, and PBS. (E) Flow cytometry analysis evaluating the specific targeting capability of Cu-EGCG@CD20 nanoparticles toward CD20-negative (5TGM1) and CD20-positive (RL) cell lines. (F) Quantitative statistical analysis of the mean fluorescence intensity derived from the flow cytometry data in (E).


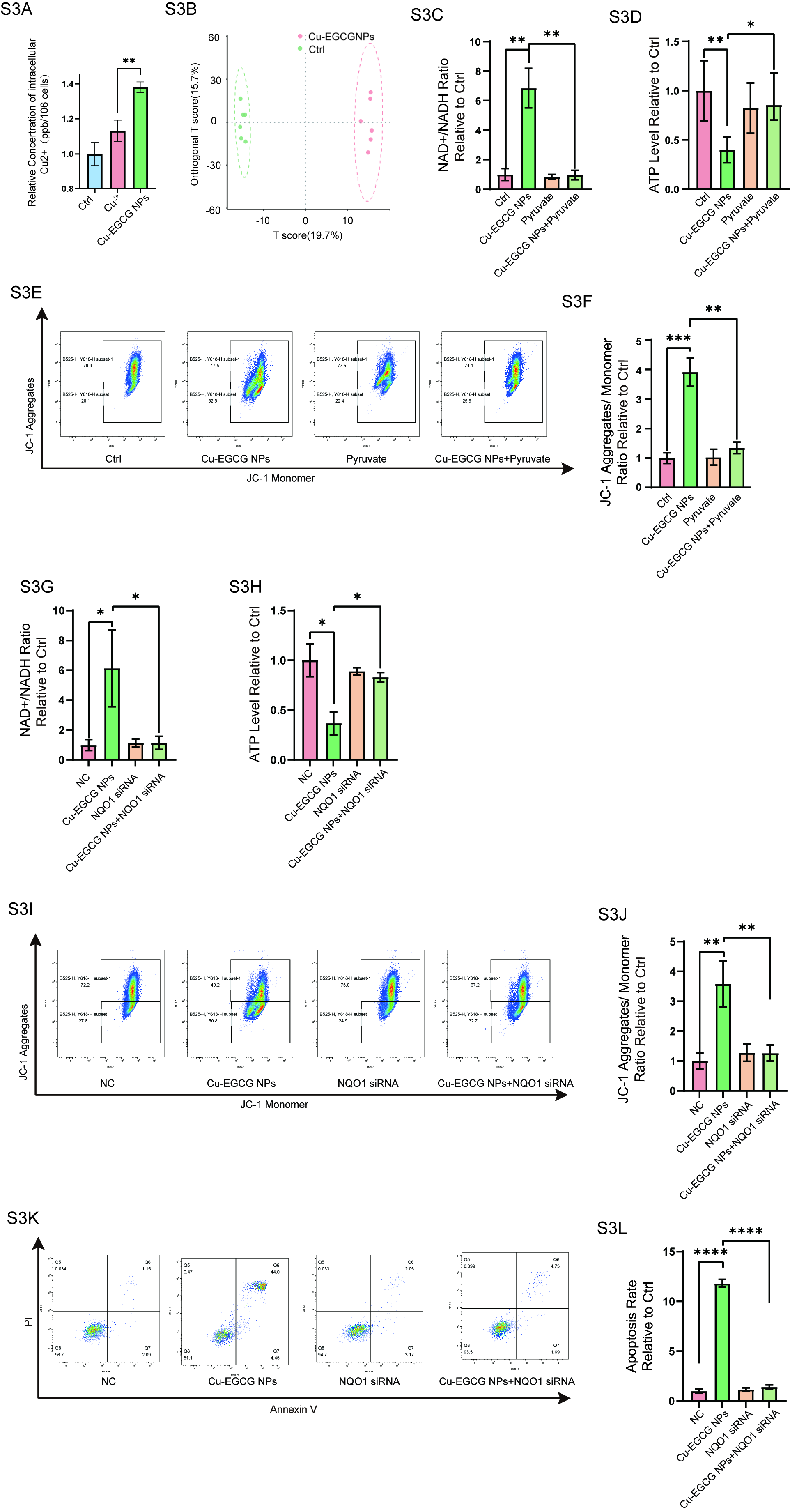


**Supplementary Figure 3. Mechanisms of metabolic reprogramming, energy crisis, and apoptosis reversal in Cu-EGCG NP-treated follicular lymphoma cells.** (A) Intracellular copper accumulation in cells treated with Cu-EGCG NPs compared to Cu²⁺-only and control groups, quantified by inductively coupled plasma mass spectrometry (ICP-MS). (B) Orthogonal Partial Least Squares-Discriminant Analysis (OPLS-DA) score plot from untargeted metabolomic profiling, demonstrating clear metabolic separation between Cu-EGCG NP-treated cells and the control group. (C, D) Quantitative analysis of the NAD⁺/NADH ratio (C) and relative intracellular ATP levels (D) in cells treated with Cu-EGCG NPs, with or without pyruvate supplementation. (E, F) Assessment of mitochondrial membrane potential using JC-1 staining in cells treated with Cu-EGCG NPs, with or without pyruvate supplementation, showing representative flow cytometry dot plots (E) and corresponding statistical quantitative analysis (F). (G, H) Quantitative analysis of the NAD⁺/NADH ratio (G) and relative intracellular ATP levels (H) in Cu-EGCG NP-treated cells following NQO1 knockdown via siRNA. (I, J) Assessment of mitochondrial membrane potential using JC-1 staining in NQO1 siRNA-transfected cells treated with Cu-EGCG NPs, displaying representative flow cytometry dot plots (I) and corresponding statistical quantitative analysis (J). (K, L) Evaluation of apoptosis by Annexin V/PI double staining in NQO1 siRNA-transfected cells treated with Cu-EGCG NPs, presenting representative flow cytometry dot plots (K) and quantitative statistical analysis of the apoptosis rate (L).


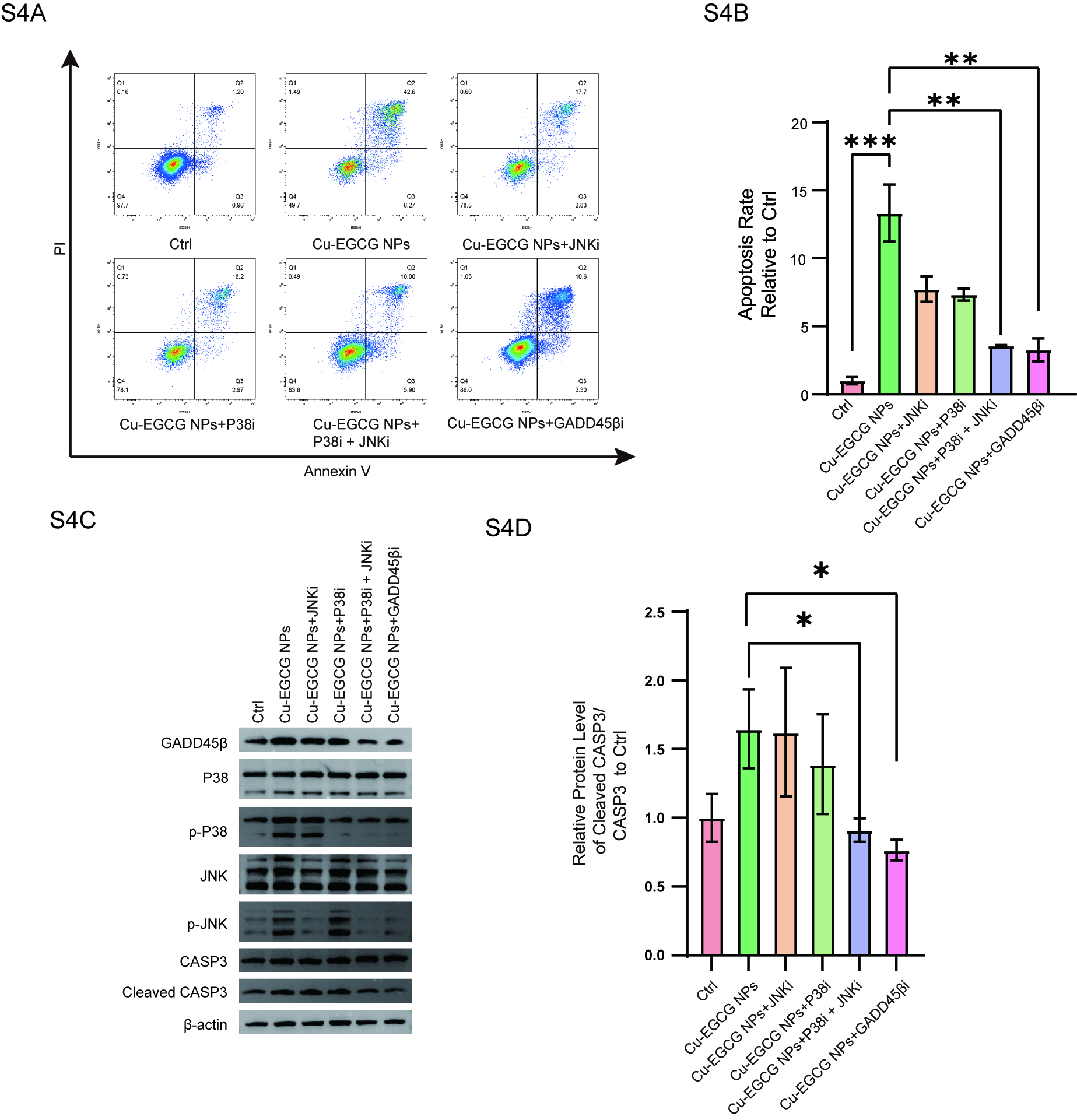


**Supplementary Figure 4. Mechanistic validation of the GADD45β-MAPK apoptotic pathway driven by Cu-EGCG nanoparticles.** (A) Representative flow cytometry dot plots evaluating apoptosis in Cu-EGCG nanoparticle-treated cells following pre-treatment with specific inhibitors for the JNK and p38 pathways, demonstrating the effective reversal of the apoptotic phenotype. (B) Quantitative statistical analysis of the apoptosis rates derived from the flow cytometry data in (A). (C) Representative Western blot analysis of p38, JNK, Caspase-3 (CASP3), and Cleaved Caspase-3 protein expression levels in cells treated with Cu-EGCG nanoparticles with or without p38 and JNK pathway inhibitors. (D) Quantitative statistical analysis of the Cleaved Caspase-3/Caspase-3 ratio based on the Western blot results in (C), confirming the terminal blockade of cell apoptosis.


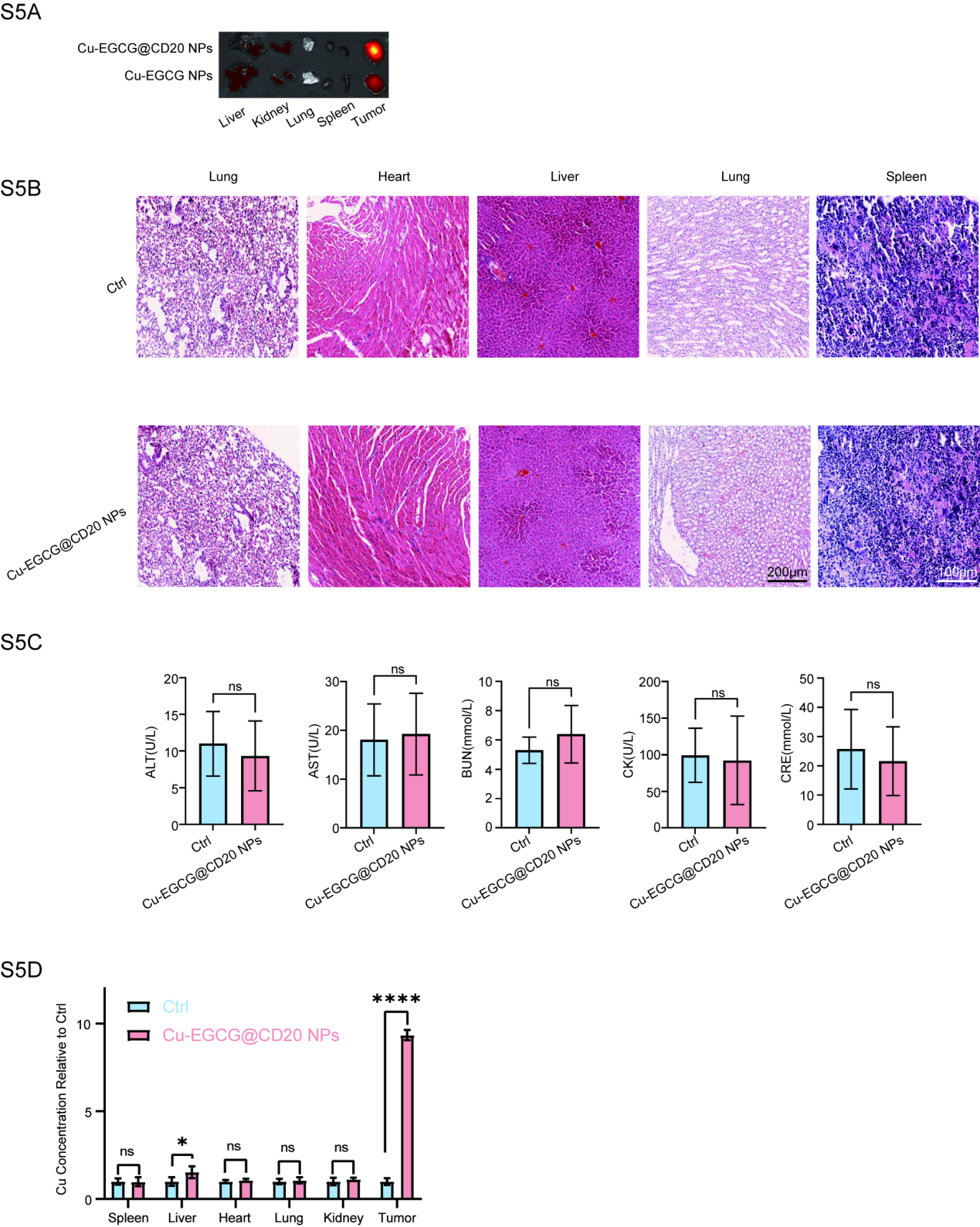


**Supplementary Figure 5. In vivo biodistribution, biosafety profile, and targeted accumulation of Cu-EGCG@CD20 nanoparticles.** (A) Ex vivo fluorescence imaging of major organs and excised tumors, tracking the biodistribution and targeted accumulation of Cy5-labeled Cu-EGCG@CD20 NPs at the study endpoint. (B) Representative hematoxylin and eosin (H&E) staining images of major organ tissue sections (heart, liver, spleen, lung, and kidney) from mice treated with Cu-EGCG@CD20 NPs, revealing no apparent pathological damage. (C) Quantitative statistical analysis of key serum biochemical parameters, including alanine aminotransferase (ALT), aspartate aminotransferase (AST), blood urea nitrogen (BUN), creatine kinase (CK), and creatinine (CRE), which all remained within normal physiological ranges. (D) Quantitative bar chart analysis of copper ion accumulation across major organs (heart, liver, spleen, lung, kidney) and tumor tissues following treatment with Cu-EGCG@CD20 NPs, measured by ICP-MS to confirm targeted tumor enrichment and biosafety.
